# Evaluation of the Septifast M^Grade^ Test on Standard Care Wards—A Cohort Study

**DOI:** 10.1371/journal.pone.0151108

**Published:** 2016-03-17

**Authors:** Franz Ratzinger, Irene Tsirkinidou, Helmuth Haslacher, Thomas Perkmann, Klaus G. Schmetterer, Dieter Mitteregger, Athanasios Makristathis, Heinz Burgmann

**Affiliations:** 1 Department of Laboratory Medicine, Division of Medical and Chemical Laboratory Diagnostics, Medical University of Vienna, Vienna, Austria; 2 Department of Medicine I, Division of Infectious Diseases and Tropical Medicine, Medical University of Vienna, Vienna, Austria; 3 Department of Laboratory Medicine, Division of Clinical Microbiology, Medical University of Vienna, Vienna, Austria; King Abdullah International Medical Research Center, SAUDI ARABIA

## Abstract

**Background:**

The immediate need for appropriate antimicrobial therapy in septic patients requires the detection of the causative pathogen in a timely and reliable manner. In this study, the real-time PCR Septifast M^Grade^ test was evaluated in adult patients meeting the systemic inflammatory response syndrome (SIRS) criteria that were treated at standard care wards.

**Methods:**

Patients with clinical suspected infection, drawn blood cultures (BC), the Septifast M^Grade^ test (SF) and sepsis biomarkers were prospectively screened for fulfillment of SIRS criteria and evaluated using the criteria of the European Centre of Disease Control (ECDC) for infection point prevalence studies.

**Results:**

In total, 220 patients with SIRS were prospectively enrolled, including 56 patients with detection of bacteria in the blood (incidence: 25.5%). BC analysis resulted in 75.0% sensitivity (95% confidence interval, CI: 61.6%– 85.6%) with 97.6% specificity (CI: 93.9%– 99.3%) for detecting bacteria in the blood. In comparison to BC, SF presented with 80.4% sensitivity (CI: 67.6%– 89.8%) and with 97.6% specificity (CI: 93.9%– 99.3%). BC and SF analysis yielded comparable ROC-AUCs (0.86, 0.89), which did not differ significantly (p = 0.558). A trend of a shorter time-to-positivity of BC analysis was not seen in bacteremic patients with a positive SF test than those with a negative test result. Sepsis biomarkers, including PCT, IL-6 or CRP, did not help to explain discordant test results for BC and SF.

**Conclusion:**

Since negative results do not exclude bacteremia, the Septifast M^Grade^ test is not suited to replacing BC, but it is a valuable tool with which to complement BC for faster detection of pathogens.

## Introduction

Sepsis is a frequent and severe condition with a high mortality rate [1]. Rapid identification of septic patients is of major importance for their survival, since they immediately need appropriate antimicrobial therapy [2]. Therefore, a timely and reliable detection of the causative pathogen(s) is essential for the proper management of septic patients. The gold standard in sepsis diagnostics is blood culture (BC) analysis, which enables antimicrobial resistance testing [3, 4]. However, a median time to a positive report of three days is necessary to detect pathogens [5, 6]. In this period of time, patients are treated with an empiric antimicrobial therapy, using broad spectrum antibiotics, which often do not appropriately cover the causative pathogens [7]. Moreover, unnecessary medication with broad spectrum antibiotics is associated with the emergence of multidrug-resistant pathogens, a higher rate of adverse drug effects as well as higher overall costs [8, 9]. Further, the sensitivity and time-to-detection of BC depends on pre-analytical factors, including administration of antimicrobial therapy before sample taking, or sufficient sample volume [10].

The Septifast M^Grade^ test (Roche Diagnostics International AG, Rotkreuz, Switzerland) is a CE (Conformité Européene) certified multiplex real-time PCR test able to detect bacterial and fungal DNA sequences of 25 pathogens directly from single whole blood sample with high specificity (98.8%) [11, 12]. In several studies, the use of such PCR tests tend to lead to an adaption of the antimicrobial therapy, a shorter length of stay in hospital, a decrement of drug side effects or a better outcome [13–18]. However, a considerable variation in the diagnostic accuracy of this test can be observed, which depends—amongst others—on the clinical setting [19]. Most studies were conducted in emergency departments or on intensive care units, neglecting standard care wards [20]. Therefore, this study aims to evaluate the diagnostic accuracy of the Septifast M^Grade^ test (SF) in SIRS patients treated on standard care wards using infection definitions of the European Centre for Disease Prevention and Control (ECDC) established for point prevalence studies [21].

## Materials and Methods

### Study design and endpoints

This prospective monocentric cohort study was conducted during a 14-month period at the Vienna General Hospital, Austria, a 2,116-bed tertiary hospital. As described before [7, 22], patients with clinically suspected sepsis on 13 surgical or 14 medical standard care wards were screened for fulfilment of at least two SIRS criteria, as defined by the ACCP/SCCM conference [23]. Clinically suspected sepsis patients were consecutively identified by screening BC requests. Iatrogenic neutropenia related to myelosuppressive therapy was not counted as a valid SIRS criterion. The study included those patients for whom BC and SF analysis were requested within a time frame of 24 hours. The study excluded patients under 18 years of age, patients with fever within 72 hours after surgical procedures, patients with HIV-positive status or patients unable to give informed consent. Prior to recruitment, written informed consent was obtained from all patients. The study was approved by the local ethics committee of the Medical University Vienna (EC-Nr: 518/2011) and conducted in accordance to the declaration of Helsinki, the Good Scientific Practice guidelines of the European Commission as well as recommendations of the STARD (Standards for Reporting of Diagnostic Accuracy) initiative [24]. After a patient’s hospital discharge, the case was reviewed using the definition criteria for hospital-acquired infection of the European Centre of Disease Control (ECDC), which include clinical, microbiological, biochemical and radiological data [21]. Bacteremia was specified as detection of bacterial species not representing contaminants in blood culture. Similarly, detection of DNAemia by applying the SF test using EDTA whole blood was considered as a means of increasing the information available. Thus, cases with a negative BC result and a positive SF result for species not representing contaminants were regarded as a true positive SF result in patients with a corresponding clinical presentation (ECDC criteria), although no vital pathogen may be present in the whole blood at the time of analysis. Potential contaminating microorganisms of the skin flora, defined according to Hall and Lyman [4], were considered as causative pathogens only when detected in at least two blood samples taken on separate occasions within the same episode. The ECDC criteria were used as a reference standard to compare the results of BC and SF analysis. According to these results, patients were allocated into six outcome groups. In non-bacteremic patients (including positive results representing contaminations per definition) the following groups were established: (1) BC negative—SF negative, (2) BC negative—SF positive, (3) BC positive—SF negative. In bacteremic patients, the groups were as follows: (4) BC negative—SF positive, (5) BC positive—SF negative, (6) BC positive—SF positive. Furthermore, the results of infection biomarkers were evaluated regarding their capacity to ascribe cases with discrepant microbiological results to true positive or true negative bacteremia. Patients that could not be allocated to outcome groups (e.g. uncertainty with respect to infection *vs* contamination in case of coagulase-negative Staphylococci (CoNS) detected in one single BC, without other evidence of infection) were also excluded from the analysis.

### Data collection

Clinical data was gathered from the medical chart at the time of study enrollment and after a patient’s hospital discharge. Blood specimens were cultured in the BacT/ALERT 3D automated BC system (bioMérieux, Marcy l'Etoile, France), utilizing sets of FA Plus (aerobic) and FN Plus (anaerobic) bottles (bioMérieux). Matrix-assisted laser desorption ionisation (MALDI) time of flight (TOF) mass spectrometry (MALDI-TOF MS) using the microflex LT together with the Biotyper database (MBT 2.2 IVD, IVD Library vers. 3.1, Bruker Daltonik GmbH, Bremen, Germany) was applied for the identification of isolates. In the case of isolates which cannot be clearly identified by MALDI-TOF MS, biochemical identification with VITEK 2 (bioMérieux), or—in the case of *Streptococcus pneumonia*—optochin susceptibility as a supplementary criterion were used. Additionally to BC, the SF was conducted according to the manufacturer’s instructions. Results obtained during thermal analysis of the probe/amplicon duplexes were analyzed automatically by the Septifast Software Set (Roche Diagnostics International AG). Further, the following infection biomarkers were evaluated: procalcitonin (PCT, Roche Diagnostics International AG, lower limit of quantification (LLOQ): 0.03 ng/ml), CRP (C-reactive protein, Beckman Coulter, Brea, USA, LLOQ: 0.04 mg/dl) and lipopolysaccharide binding protein (LBP, Siemens Healthcare, Erlangen Germany, LLOQ: 0.8 μg/ml). White blood cell counts (WBC) were analyzed on a Sysmex XE-2100 or XE-5000 (Sysmex, Kobe, Japan; LLOQ: not provided). All infection biomarkers were assessed within 18 hours of the BC request. No blinding techniques were applied to mask results of BC, SF or laboratory parameters. All laboratory work was accomplished in an ISO 9001:2008 certified medical laboratory. Raw data cannot be made openly available to protect the privacy of participants. Further information about the data and conditions for access to anonymized (de-identified) raw data can be requested from the corresponding author.

### Statistical analysis

Data were statistically analyzed using SPSS 21.0 (IBM, Hercules, USA) and MedCalc (Version 14.8.1, MedCalc Software bvba, Ostend, Belgium). Data following a Gaussian distribution (assessed using the Shapiro-Wilk test) are presented as mean ±standard deviation (SD) and analyzed using the Student's t-test or ANOVA with Dunnett’s test for post-hoc comparisons. Other continuous data are given as the median and interquartile range (Q1–Q3) and analyzed with the Mann-Whitney U-test or the Kruskal-Wallis test. Categorical parameters are described by counts and percentages and analyzed using the generalized Fisher exact test. Further, for the BC and SF evaluation, ROC-AUC (receiver operating characteristics—area under the curve) analysis was conducted and the results were compared by applying the DeLong test. The diagnostic performances of BC and SF are given by the sensitivity, the specificity, the negative and positive likelihood ratio (LR^-^, LR^+^) as well as the negative and positive predictive value (NPV, PPV). Their 95% confidence intervals were computed by applying a bootstrapping procedure (n = 2000). Statistical significance was defined as p-values less than 0.05 (two-sided). The Bonferroni-Holm method was applied to correct for an increase of the type I error probability related to multiple testing.

## Results

### Demographic characteristics of the study population

**Fig 1:**
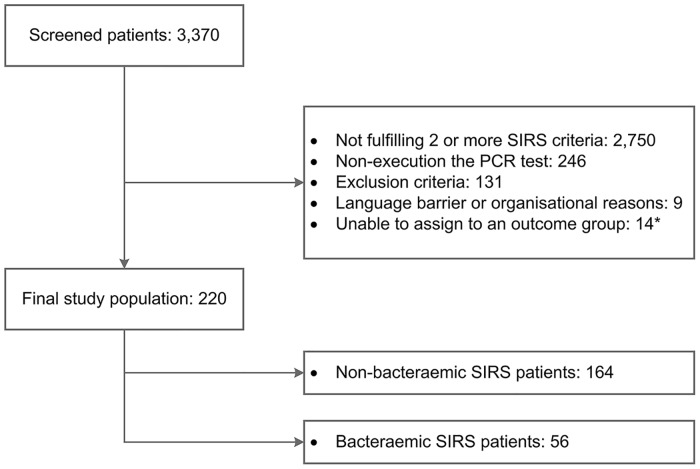
Patient recruitment process of the study population. *including cases with *coagulase-negative Staphylococci* in a single blood culture analysis without any evidence of infection.

Between July 2011 and September 2012, a total of 3,370 patients with suspected sepsis were screened. Of these, 2,750 patients fulfilled less than two SIRS criteria and 140 patients met exclusion criteria. After study inclusion, 14 patients were excluded that could not be allocated to the proposed outcome groups. In total, 220 SIRS patients were enrolled in this study. Fig 1 presents the recruitment process of the final study cohort.

According to the ECDC criteria applied, 149 SIRS patients (67.7%) suffered from an infection and 71 patients (32.3%) suffered from SIRS due to a non-infectious cause. The most prevalent causes of non-infectious SIRS were hematological malignancies (disease or treatment-related, 33.8%), solid organ malignancies (19.7%), auto-immune diseases (14.1%) and bleeding or embolism (7%). The most frequent infections were pneumonia (n = 44, 29.5%), urinary tract infections (n = 22, 14.8%) and gastrointestinal system infections (n = 21, 14.1%). S1 Table presents the distribution of ECDC classes of patients with infections to make more the percentages of infections understandable. In DNAemic BC-/SF+ cases, special attention was given to the fulfillment of the corresponding ECDC criteria. In one patient with endocarditis (echocardiographic and histological confirmation), *Streptococcus spp*. was detected by the SF test, while six sets of BC analyses remained negative. Due to the PCR result, the antibiotic therapy was changed to Penicillin G, which led to a substantial increase of the patient’s status and to a normalization of CRP. Therefore, this case was regarded as a true positive SF result. Among the study population, eight patients were suffering from endocarditis (3.6%).

In total, (true positive) pathogens detected in blood specimens were found in 56 patients (25.5%). The most common sources were gastrointestinal system infections (n = 12, 21.4%), an unknown infection site (n = 10, 17.9%), as well as catheter-related infections (n = 8, 14.3%). S2 Table displays the pathogens detected by BC and SF. Within patients with a positive BC or SF result, Gram-positive pathogens were found in 26 participants (46.4%), whereas Gram-negative pathogens were detected in 25 cases (44.6%). In further five patients (8.9%), Gram-positive pathogens were detected together with Gram-negative bacteria (n = 4) or with fungi (n = 1). The most prevalent pathogens detected were *Staphylococcus aureus* and *Escherichia coli* (both: n = 11, individual cases). Table 1 presents data regarding the distribution of pathogens in the outcome groups.

**Table 1:** Distribution of detected pathogens.

Group	Pathogens
***no bacteremia***	
-**BC-/SF-** n = 156 (70.9%)	none
-**BC-/SF+** n = 4 (1.8%)	CoNS (n = 4)
-**BC+/SF-** n = 4 (1.8%)	CoNS (n = 4)
***detected pathogens in blood specimens***	
- **BC-/SF+** n = 14 (6.4%)	*Klebsiella pneumoniae/oxytoca* (n = 5), *Staphylococcus aureus* (n = 2)^1^, *Enterococcus faecium* (n = 2), *Streptococcus pneumoniae* (n = 2), *Streptococcus species* (n = 1), *Pseudomonas aeruginosa* (n = 1), *Enterobacter cloacae/aerogenes* (n = 1)
- **BC+/SF-** n = 11 (5.0%)	*Escherichia coli* (n = 3), *Staphylococcus aureus* (n = 2), *Staphylococcus epidermidis* (n = 1), *Citrobacter koseri* (n = 1), *Klebsiella pneumoniae* (n = 1), *Pseudomonas aeruginosa* (n = 1), *Raoultella ornithinolytica* (n = 1), *Streptococcus mutans* (n = 1)
- **BC+/SF+** n = 31 (14.1%)	*Staphylococcus aureus* (n = 7)^1^, *Escherichia coli* (n = 5)^2^, *Pseudomonas aeruginosa* (n = 4)^3^, *Klebsiella pneumoniae/oxytoca* (n = 3)^4^, *CoNS* (n = 3), *Enterobacter cloacae/aerogenes* (n = 2)^5^, *Enterococcus faecalis* (n = 2)^6^, *Candida albicans* (n = 1)^7^, *Enterococcus faecium* (n = 1), *Streptococcus pyogenes* (n = 1), *Streptococcus mitis* (n = 1), *Streptococcus constellatus* (n = 1)

BC = blood culture, SF = Septifast M^Grade^ test,

[–] negative,

[+] positive,

*detected in a localized CNS infection, excluded in comparison analysis,

^1^one cases with additional CoNS in BC and SF,

^2^one case with additional *Staphylococcus epidermidis* in BC,

^3^one case with additional detection of *Enterococcus faecalis* in SF and E. coli in BC,

^4^one case with polymicrobial infection: *Escherichia coli* (SF and BC), *Bacteroides thetaiotaomicron* (BC), Corynebacterium species (BC),

^5^one case with additional detection of *Citrobacter freundii* in BC,

^6^one case with additional detection of *Escherichia coli* ESBL (BC),

^7^additonal detection of Streptococcus pneumoniae in SF

Among the 220 patients, 127 patients (57.7%) were males and 93 (42.3%) were females. Participants had a median age of 56.5 years (41.5–68.0) and a median BMI of 25.3 (21.5–29.0). The median length of hospital stay was 15.5 days (9.0–27.5 days). Within the study population, 187 patients (85.0%) were treated on a medical standard care ward and 33 patients (15.0%) were treated on surgical standard care wards. Of the 220 patients, 93 (42.3%) presented with two SIRS criteria, 108 patients (49.1%) with three and 19 patients (8.6%) with four. No significant differences were found in patients’ characteristics or in the individual (numerically scaled) SIRS parameters between SIRS patients with positive BC or SF test and those having both tests negative. Among the study population, 45.5% patients (n = 100) suffered from a neoplasm and a large proportion presented with leukopenia (47 patients, 21.4%, < 4 G/L WBC count). Table 2 presents the clinical and epidemiological data of the study cohort.

**Table 2:** Patient characteristics of the study population and their distribution between non-bacteremic and bacteremic participants.

	n	All	BC- and SF-	BC+ and/or SF+	p-value
**Male**	220	127 (57.7%)	92 (56.1%)	35 (62.5%)	0.436
**Wards medical**	220	187 (85%)	142 (86.6%)	45 (80.4%)	0.281
**Antibiotics before BC taken**	220	50 (22.7%)	40 (24.4%)	10 (17.9%)	0.360
SIRS criteria*	220	93:108:19	68:79:17	25:29:2	0.306
**Catheter**	223	52 (23.6%)	35 (21.3%)	17 (30.4%)	0.281
**Neoplasm**	220	100 (45.5%)	71 (43.3%)	29 (51.8%)	0.281
**In-hospital mortality**	220	28 (12.7%)	18 (11.0%)	10 (17.9%)	0.244
**Age**	220	56.5 (41.5–68.0)	56 (40.0–67.0)	57.5 (45.0–68.0)	0.284
**BMI**	220	25.3 (21.5–29.0)	25.3 (21.6–29.2)	25.4 (21.0–27.8)	0.413
**Respiration rate**	216	21.0 (16.0–24.0)	21(16.0–24.0)	21 (16.0–24.0)	0.722
**BT**	220	38.5 (38.1–39.0)	38.5 (38.1–39.0)	38.8 (38.1–39.2)	0.109
**Heart rate**	220	100.0 (92.0–110.0)	100.0 (92.5–110.0)	100.0 (88.5–110.0)	0.772
**LOS**	220	15.5 (9.0–27.5)	15.5 (8.5–27.5)	16 (10.0–28.5)	0.465
**WBC**	220	10.29 (5.2–15.3)	10.2 (4.9–15.1)	10.5 (6.2–16.1)	0.592
**IL-6**	216	49.5 (22.3–15.2)	46.4 (21.1–109.0)	57.5 (32.4–172.6)	0.058
**PCT**	217	0.36 (0.15–1.45)	0.27 (0.13–0.73)	1.79 (0.41–4.8)	<0.001**
**CRP**	213	14.7 (9.0–22.0)	13.7 (8.5–20.3)	15.9 (9.4–25.2)	0.027
**LBP**	218	27.0 (18.6–40.3)	25.8 (17.1–38.4)	34.2 (21.2–52.2)	0.004**

*2:3:4 SIRS criteria, BC = blood culture, BMI = body mass index, BT = body temperature, WBC = white blood count, LOS = length of hospital stay, IL-6 = interleukin 6, CRP = C-reactive protein, LBP = lipopolysaccharide binding protein,

**statistically significant after application of the Bonferroni-Holm method

### Evaluation of the test performance

Amongst the study population, 31 patients (14.1%) had a concordant positive result in BC as well as in SF. In 156 patients (70.9%), concordant negative results were found in both analyses. Discordance between both tests was found in 33 patients (15.0%).

The blood samples of 18 participants provided a positive SF result, while the BC remained negative and in 15 patients the BC analysis was positive while the SF yielded a negative test result. Among the patients with detected pathogens in blood specimens (true positives), there were 42 participants (75.0%) with a positive BC and 45 patients (80.4%) with a positive SF result. 160 patients in BC and also 160 participants in SF were found to be true negatives. False negative results were found in 14 patients by BC and in further 11 patients by SF. Four patients were false positive in BC only, with CoNS and a further four patients were false positive in SF with CoNS. Within the true positive SF results with negative BC, *Klebsiella pneumoniae/oxytoca* (n = 5), *Enterococcus faecium*, *S*. *aureus and Streptococcus pneumoniae* (each: n = 2) were most commonly detected. Additionally, S3 Table presents data of SF+/BC- cases. Within the patients with true positive BC and negative SF results, *E*. *coli* (n = 3) and *S*. *aureus (n = 2)* were most frequently isolated (Table 1).

**Fig 2:**
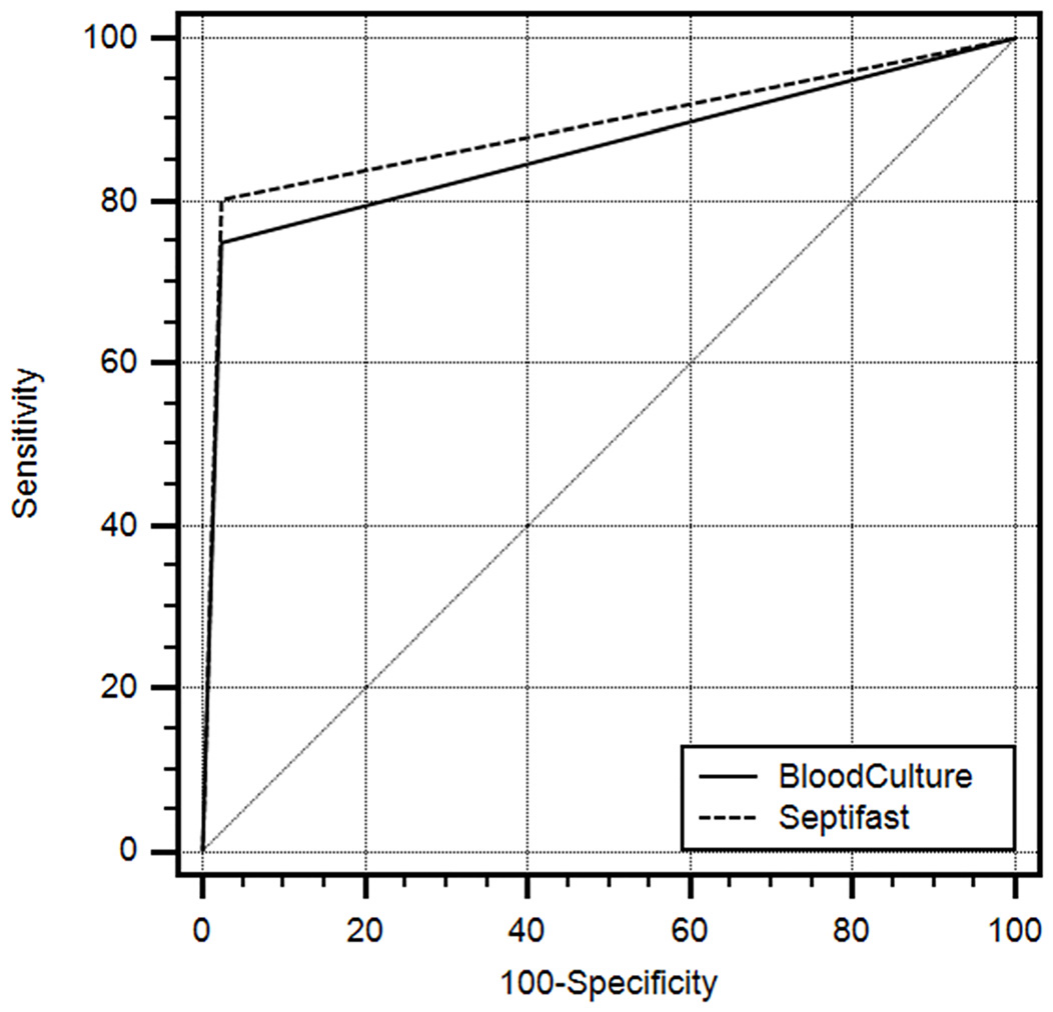
ROC-AUC curves of blood culture (BC) and SF analysis. BC: 0.863ROC-AUC (CI: 0.810–0.905), SF: 0.890 ROC-AUC (CI: 0.841–0.928); the difference between both ROC-AUCs was not found to be statistically significant (p = 0.558 DeLong test).

BC analysis resulted in 75.0% sensitivity with 97.6% specificity and SF resulted in 80.9% sensitivity with 97.6% specificity (see: Table 3). BC yielded 0.863 ROC-AUC (CI: 0.810 to 0.905, Fig 2) and SF 0.890 ROC-AUC (CI: 0.841 to 0.928). The difference between both ROC-AUCs was not found to be statistically significant (p = 0.558).

**Table 3:** Diagnostic outcome measures of BC and SF analysis.

	BC		SF	
	negative	positive	negative	positive
**No Bacteremia**	160	4	160	4
**Bacteremia**	14	42	11	45
**Sensitivity**	75.0% (61.6%– 85.6%)		80.4% (67.6%– 89.8%)	
**Specificity**	97.6% (93.9%– 99.3%)		97.6% (93.9%– 99.3%)	
**AUC**	86.3% (81.0%– 90.5%)		89.0% (84.1%– 92.8%)	
**LR**^**+**^	30.8 (11.5–81.9)		33.0 (12.4–87.5)	
**LR**^**-**^	0.26 0.20–0.40		0.20 0.10–0.30	
**PPV**	91.3 (79.2–97.6)		91.8 (80.4–97.7)	
**NPV**	92.0 (86.9–95.5)		93.6 (88.8–96.7)	

bacteremia was defined according to the codebook of the PPS survey of the ECDC (21) BC: blood culture analysis, SF: polymerases chain reaction analysis (here: Septifast M^GRADE^ test, Roche), 95% confidence intervals are given in brackets, LR^+^: positive likelihood ratio, LR^-^: negative likelihood ratio, PPV: positive predictive value, NPV: negative predictive value;

In non-bacteremic patients with positive BC and negative SF, the mean time to positivity was 37.75 hours (±39.1 hours). In bacteremic patients with positive BC and negative SF the mean time to positivity was 15.7 hours (±12.5 hours) and in bacteremic patients having a positive result in both tests, the mean time to positivity was 15.8 hours (±11.4 hours). These differences were found to be statistically significant (ANOVA, p = 0.031). In post-hoc analysis, bacteremic patients for whom both tests were positive differed significantly from non-bacteremic patients with positive BC (p = 0.026), but not from bacteremic patients with negative SF (p = 0.999).

### Performance of sepsis biomarkers

For discriminating between SIRS patients with positive and negative BC/SF test, PCT was the best parameter (p<0.001) with a median of 0.27 ng/ml (Q1–Q3: 0.13–0.73) in non- bacteremic SIRS patients and 1.79 ng/ml (Q1–Q3: 0.41–4.78) in bacteremic SIRS patients with detected pathogens. When using a cut-off value of 0.37 ng/ml pre-defined in the literature [25], 35 patients with a positive SF test and also 35 patients with a positive BC were found in the higher PCT group (p<0.001). Nevertheless, if in 113 patients with low PCT levels the SF analysis had not been conducted accordingly, ten patients with a true positive SF test result and eight patients with a true positive BC would have been missed. S4 Table presents data of the 13 patients with PCT levels <0.37 ng/ml.

Further, LBP and CRP levels differed significantly between bacteremic and non-bacteremic patients (p = 0.001, p = 0.027, Table 2). PCT was the only parameter with a significant difference in the distribution between the six outcome groups analyzed (p<0.001, see Table 4). In post-hoc analysis, only non-bacteremic patients with negative BC and negative SF results significantly differed from bacteremic patients for whom both tests were positive (p<0.001) or only BC positive (p = 0.027).

**Table 4:** Distribution of sepsis biomarkers within the six outcome groups.

		No bacteremia			Bacteremia			
Parameter	n	BC-/SF-	BC-/SF+	BC+/SF-	BC-/SF+	BC+/SF-	BC+/SF+	p-value^1^
**PCT**	224	0.27 (0.13–0.73)	0.23 (0.14–1.03)	0.39 (0.17–1.10)	0.65 (0.16–4.78)	4.26 (0.38–9.49)	1.87 (0.62–3.43)	<0.001
**LBP**	225	25.3 (16.6–38.4)	28.9 (23.6–31.5)	38.4 (23.9–51.0)	24.9 (20.6–64.7)	36.9 (22.0–61.9)	36.0 (21.8–50.4)	0.058
**CRP**	220	13.9 (8.5–20.3)	13.7 (13.4–14.3)	18.6 (8.6–28.5)	17.6 (8.7–31.9)	18.8 (10.3–21.9)	15.6 (10.3–26.0)	0.381
**IL-6**	223	46.4 (21.1–110.4)	40.3 (18.2–51.3)	48.8 (26.6–73.3)	60.4 (32.4–222.1)	51.4 (44.4–172.6)	60.8 (22.5–170.5)	0.466

BC = blood culture, SF = Septifast M^Grade^ test,

[–] negative,

[+] positive,

^1^Global test: Kruskal-Wallis test; post-hoc testing applied for PCT using nonparametric Dunn´s test (implemented in Graph Pad Prism; No bacteremia BC-/SF- vs. No bacteremia BC-/SF+: p = > 0.999, No bacteremia BC-/SF- vs No bacteremia BC+/SF-: p = > 0.999, No bacteremia BC-/SF- vs. Bacteremia BC-/SF+: p = 0.342, No bacteremia BC-/SF- vs. Bacteremia BC+/SF-: p = 0.0267, No bacteremia BC-/SF- vs. Bacteremia BC+/SF+ p< 0.001.

## Discussion

The aim of the study was to evaluate the Septifast M^Grade^ test in adult patients with suspected sepsis treated on the standard care ward in a tertiary hospital. Prior to this undertaking, various studies have been performed including patients in the emergency department or on the intensive care unit, neglecting standard care wards. Since a relevant proportion of septic patients are treated on standard care wards, with a major economic impact, and many physicians on these wards are not specialized in the management of infectious diseases, these patients warrant special attention. To increase the pre-test probability of bacteremia and to focus on a relevant patient cohort, exclusively patients fulfilling two or more SIRS criteria were included in this study. In total, 220 SIRS patients including 56 patients with detected pathogens in blood specimens (= 25.5% prevalence) were prospectively enrolled. Related to the collection site, participants were rather young (in median: 56.5 years), with a high rate of neoplasms (45.5%) and a high rate of catheters (23.6%), which elevated the rate of *S*. *epidermidis* (12.5%) detected in blood specimens. These pathogens were considered as true positives when detected in more than one independent blood draw. No significant differences in patients’ characteristics were found, indicating an appropriate matching of non-bacteremic SIRS patients to SIRS patients with detected pathogens. Further, no bacteremic BC-/SF+ case was found that did not fulfill the corresponding ECDC infection criteria. BC yielded 75.0% sensitivity and 97.6% specificity for detecting bacteria in blood specimens, whereas SF presented a higher sensitivity and with a similar specificity (80.4%, 97.6% respectively). In ROC-curve analysis, both tests resulted in a comparable ROC-AUC (0.863 vs. 0.890, p = 0.558).

In comparison to the literature, we found a better performance of the SF test. Two meta-analyses on the diagnostic performance of the Septifast MGrade test are published. Chang et al. published a meta-analysis determining 75% sensitivity (95% CI: 65%–83%) and 92% specificity (90%–95%) for detecting bacteremia or fungemia [19]. In a recent meta-analysis by Dark et al., SF showed 68% sensitivity (63%–73%) and 86% specificity (84%–89%) [20]. Some methodological differences between both meta-analyses exist, which might clarify these differences in the outcome results. The analysis of Dark et al. restricted the analysis to those publications using exclusively BC as the reference standard, whereas Chang et al. also accepted publications with clinically documented infections and various reference standards. The prevalence (pre-test probability) is not taken into account for calculating these diagnostic measures, but it cannot be ruled out that the clinical setting might have had a major impact on their results. In the meta-analyses, the cumulative proportion of bacteremia/fungemia was 0.23 [19] and 0.17 [20] respectively, while in our case the bacteremia proportion of patients with detected pathogens was 0.26.

In patients with a corresponding clinical presentation and with clinical improvement in the course of an antibiotic therapy, a positive SF test was also considered a true positive result in cases with negative BC analysis. We are aware that this procedure has an impact on the test performance, but we do not see any malfunction of the SF test in such patients. In addition, an improved “gold standard” index test would be highly desirable to avoid such uncertainties.

Subsequently, the higher pre-test probability might result in a higher rate of true positives in SF analysis. In the literature, as well as in our study, SF presented a higher specificity than sensitivity, implying its clinical utility as a rule in-test rather than a rule-out test for bacteremia. Therefore, the SF analysis is not recommendable as a screening test. Between BC and SF analysis, a discordance rate of 15.0% (n = 33) was found. In BC analysis, pathogens were isolated in 11 patients, while SF remained negative. Amongst those patients were nine with pathogens detectable within the spectrum of the SF assay. The causative pathogen was detected by SF in 14 patients (25.0%), while BC remained negative, indicating a substantial benefit for these patients. Up to a rate of 32.2% positive SF tests with negative BC results is reported in the literature [14, 26]. One might speculate about the reasons for the heterogeneity within the rates of SF positive BC negative results. Apart of the evaluation design, disease severity (analysis was restricted to SIRS patients) might be an influencing factor for the higher true positive rate of SF. Since in BC the detection of pathogens is restricted to vital microorganisms, SF analysis offers the potential advantage of detecting the DNA of non-vital pathogens. In accordance, in the literature there is some evidence for greater diagnostic accuracy of SF compared to BC in patients with antibiotic treatment before sample collection [15, 27]. In our study, no significant difference was found in the detection rate between those with or without antimicrobial therapy before BC was taken (SF p = 0.235, BC: p = 0.125). The analysis of time-to-positivity of BC did not show a trend for BC to turn positive faster in those patients with concordantly positive SF than in those with a negative SF result. Such a trend would have indicated a higher pathogen load in blood specimens of SF positive bacteremic patients. However, there are a plethora of PCR inhibitors, including collagen, IgG, myoglobin, heparin or antiviral substances, which might have an inhibitory influence on the test SF results [28]. On the clinical level, a comprehensive evaluation of such inhibitory substances on PCR tests is yet to be conducted.

The assessment of sepsis biomarkers showed that SIRS patients with detected pathogens in blood specimens had elevated PCT, LBP and CRP levels compared to non-bacteremic SIRS patients. Within the laboratory parameters tested, PCT presented the best potency to discriminate between non-bacteremic SIRS patients and those with detected pathogens. In the literature, a high concordance between elevated PCT levels and positive BC or SF results are described [15, 16, 29]. Subsequently, PCT was proposed to be used as a screening parameter indicative for the utility of subsequently ordered for BC/SF investigations. If a pre-defined cut-off value of 0.37 ng/ml [25] had been used, 13 patients with true positive SF result and ten bacteremic patients with a positive BC result would have been missed. There is evidence that the PCT expression levels are influenced by the causing pathogen type and by the patient´s infection site. Especially Gram-positive pathogens as well as conditions like endocarditis cause a lower PCT expression [30–32]. Moreover, higher PCT levels are also found in patients with malignancies, after surgical procedures, myocardial infarction or hemodialysis [33–38]. Although PCT is a useful marker in sepsis diagnostics and therapy monitoring [39], our data would not support such a triaging based on the PCT result. Furthermore, an additional discriminatory value of these biomarkers was not found for patients with a discordant SF to BC result.

Several limitations must be disclosed in this monocentric cohort study. The BC and SF analysis was requested by the physician in charge according to the patient’s clinical presentation. Along with the fulfilment of two or more SIRS criteria, availability of both analyses was the primary selection criterion in this study. Therefore an observational bias cannot be ruled out. Moreover, a timeframe of 24 hours between blood culture, SF or biomarker taking was accepted. Within this timeframe antibiotic treatment might have provoked a discordant result. There is no clear data concerning the clearance of pathogenic DNA in the human blood circulation. In a prospective observational study including 51 critically ill patients suffering from *Acinetobacter baumannii* infection, the mean bacterial load was initially 2.15 log copies per mL [40]. The bacterial clearance rate was 0.088 log copies per mL per day, which might indicate that our accepted time lag is of minor influence for the total detectable bacterial DNA load. Further, antibiotic treatment prior to sample collection could have impacted on the false negative rate of both tests. Furthermore, there is evidence showing that the expression of biomarkers is the highest on the third day after onset of sepsis [41, 42]. However, a delay in taking specimens for diagnostics and antibiotic pre-treatment are common issues in daily practice and are therefore not seen as major limitations.

A new generation of microbiological multiplex PCR tests is upcoming, using an electrospray ionization-mass spectrometry after the polymerase chain reaction followed by (PCR/ESI-MS). These tests might have an improved sensitivity and are able to detect a limited range of antibiotic resistance marker [43, 44].

A major advantage of the SF is a shorter time-to-result, which may lead to a faster modification of the empirical antimicrobial therapy [45, 46]. Further, the prescription practice of antimicrobial therapy may change, since waiting for microbiological test results could improve the outcome for patients [47]. In our laboratory as well as in most other centers the SF assay is applied on samples arriving in the laboratory at working days before midday for a same day result. A time-to-result analysis was left undone, since an observational bias would have been created in relation to our inclusion criteria (24 hours’ timeframe between BC and SF). In the literature, a mean time of 15 to 18 hours is necessary to obtain a result from SF analysis [48, 49]. Thus, a simplified application of the assay, e.g. in form of a cartridge based POCT-test, would be highly desirable. Moreover, detection of resistance genes is currently not included in SF, and therefore this test cannot replace the BC but can be recommended as a helpful add-on in patients with a high need of a fast pathogen detection.

## Conclusion

The specificity of SF is in a higher range than its sensitivity. Since negative results do not exclude bacteremia, the Septifast M^Grade^ test is not suited to replacing BC, but it is a valuable tool with which to complement BC for faster detection of pathogens.

## Supporting Information

S1 Table Distribution of infections according to ECDC classification criteria. (DOCX)

S2 Table Comparison between BC and SF analysis. (DOCX)

S3 Table BC negative patients with detection of pathogens in the SF test. (DOCX)

S4 Table Patients with detected pathogens and <0.37ng/ml PCT levels. (DOCX)

## References

[1] Angus DC, Wax RS. Epidemiology of sepsis: an update. Critical care medicine. 2001 7;29(7 Suppl):S109–16. 11445744 10.1097/00003246-200107001-00035

[2] Kumar A, Roberts D, Wood KE, Light B, Parrillo JE, Sharma S, et al Duration of hypotension before initiation of effective antimicrobial therapy is the critical determinant of survival in human septic shock. Critical care medicine. 2006 6;34(6):1589–96. 16625125 10.1097/01.CCM.0000217961.75225.E9

[3] Mancini N, Carletti S, Ghidoli N, Cichero P, Burioni R, Clementi M. The Era of Molecular and Other Non-Culture-Based Methods in Diagnosis of Sepsis. Clin Microbiol Rev. 2010 1 1, 2010;23(1):235–51. 10.1128/CMR.00043-09 PMC2806664 20065332

[4] Hall KK, Lyman JA. Updated review of blood culture contamination. Clin Microbiol Rev. 2006 10;19(4):788–+. 17041144 10.1128/CMR.00062-05 PMC1592696

[5] Westh H, Lisby G, Breysse F, Boddinghaus B, Chomarat M, Gant V, et al Multiplex real-time PCR and blood culture for identification of bloodstream pathogens in patients with suspected sepsis. Clinical microbiology and infection: the official publication of the European Society of Clinical Microbiology and Infectious Diseases. 2009 6;15(6):544–51. 10.1111/j.1469-0691.2009.02736.x 19392905

[6] Bloos F, Sachse S, Kortgen A, Pletz MW, Lehmann M, Straube E, et al Evaluation of a polymerase chain reaction assay for pathogen detection in septic patients under routine condition: an observational study. PloS one. 2012;7(9):e46003 10.1371/journal.pone.0046003 23029360 PMC3459981

[7] Ratzinger F, Eichbichler K, Schuardt M, Tsirkinidou I, Mitteregger D, Haslacher H, et al Sepsis in standard care: patients' characteristics, effectiveness of antimicrobial therapy and patient outcome-a cohort study. Infection. 2015 4 4. 10.1007/s15010-015-0771-0 25840554

[8] Hanon FX, Monnet DL, Sorensen TL, Molbak K, Pedersen G, Schonheyder H. Survival of patients with bacteraemia in relation to initial empirical antimicrobial treatment. Scandinavian journal of infectious diseases. 2002;34(7):520–8. . Epub 2002/08/28. eng. 12195878 10.1080/00365540110080827

[9] Rao GG. Risk factors for the spread of antibiotic-resistant bacteria. Drugs. 1998 3;55(3):323–30. 9530540 10.2165/00003495-199855030-00001

[10] Willems E, Smismans A, Cartuyvels R, Coppens G, Van Vaerenbergh K, Van den Abeele A-M, et al The preanalytical optimization of blood cultures: a review and the clinical importance of benchmarking in 5 Belgian hospitals. Diagnostic Microbiology and Infectious Disease. 2012;73(1):1–8. 10.1016/j.diagmicrobio.2012.01.009 22578933

[11] Vince A, Lepej SZ, Barsic B, Dusek D, Mitrovic Z, Serventi-Seiwerth R, et al LightCycler SeptiFast assay as a tool for the rapid diagnosis of sepsis in patients during antimicrobial therapy. Journal of medical microbiology. 2008 10;57(Pt 10):1306–7. 10.1099/jmm.0.47797-0 18809565

[12] Lehmann LE, Hunfeld KP, Emrich T, Haberhausen G, Wissing H, Hoeft A, et al A multiplex real-time PCR assay for rapid detection and differentiation of 25 bacterial and fungal pathogens from whole blood samples. Medical microbiology and immunology. 2008 9;197(3):313–24. 18008085 10.1007/s00430-007-0063-0

[13] Lodes U, Meyer F, Konig B, Lippert H. [Microbiological sepsis screening in surgical ICU patients with the "lightCycler" Septifast test—a pilot study]. Zentralblatt fur Chirurgie. 2009 6;134(3):249–53. 10.1055/s-0028-1098776 19536720

[14] Grif K, Fille M, Wurzner R, Weiss G, Lorenz I, Gruber G, et al Rapid detection of bloodstream pathogens by real-time PCR in patients with sepsis. Wiener klinische Wochenschrift. 2012 4;124(7–8):266–70. 10.1007/s00508-012-0159-4 22527822

[15] Pasqualini L, Mencacci A, Leli C, Montagna P, Cardaccia A, Cenci E, et al Diagnostic performance of a multiple real-time PCR assay in patients with suspected sepsis hospitalized in an internal medicine ward. Journal of clinical microbiology. 2012 4;50(4):1285–8. 10.1128/JCM.06793-11 22322348 PMC3318564

[16] Mauro MV, Cavalcanti P, Perugini D, Noto A, Sperli D, Giraldi C. Diagnostic utility of LightCycler SeptiFast and procalcitonin assays in the diagnosis of bloodstream infection in immunocompromised patients. Diagnostic microbiology and infectious disease. 2012 8;73(4):308–11. 10.1016/j.diagmicrobio.2012.04.006 22626731

[17] Avolio M, Diamante P, Zamparo S, Modolo ML, Grosso S, Zigante P, et al Molecular identification of bloodstream pathogens in patients presenting to the emergency department with suspected sepsis. Shock. 2010 7;34(1):27–30. 10.1097/SHK.0b013e3181d49299 20090568

[18] Lehmann LE, Alvarez J, Hunfeld KP, Goglio A, Kost GJ, Louie RF, et al Potential clinical utility of polymerase chain reaction in microbiological testing for sepsis. Critical care medicine. 2009 12;37(12):3085–90. 10.1097/CCM.0b013e3181b033d7 19633541

[19] Chang SS, Hsieh WH, Liu TS, Lee SH, Wang CH, Chou HC, et al Multiplex PCR system for rapid detection of pathogens in patients with presumed sepsis—a systemic review and meta-analysis. PloS one. 2013;8(5):e62323 10.1371/journal.pone.0062323 23734173 PMC3667030

[20] Dark P, Blackwood B, Gates S, McAuley D, Perkins GD, McMullan R, et al Accuracy of LightCycler((R)) SeptiFast for the detection and identification of pathogens in the blood of patients with suspected sepsis: a systematic review and meta-analysis. Intensive care medicine. 2015 1;41(1):21–33. 10.1007/s00134-014-3553-8 25416643

[21] European Centre for Disease Prevention and Control. Point prevalence survey of healthcare-associated infections and antimicrobial use in European acute care hospitals—protocol version 4.3. Stockholm: ECDC; 2012; Available from: http://ecdc.europa.eu/en/publications/publications/0512-ted-pps-hai-antimicrobial-use-protocol.pdf. [Assessed: 31.08.2015]

[22] Ratzinger F, Schuardt M, Eichbichler K, Tsirkinidou I, Bauer M, Haslacher H, et al Utility of sepsis biomarkers and the infection probability score to discriminate sepsis and systemic inflammatory response syndrome in standard care patients. PloS one. 2013;8(12):e82946 10.1371/journal.pone.0082946 24349403 PMC3859603

[23] Bone RC, Balk RA, Cerra FB, Dellinger RP, Fein AM, Knaus WA, et al Definitions for sepsis and organ failure and guidelines for the use of innovative therapies in sepsis. The ACCP/SCCM Consensus Conference Committee. American College of Chest Physicians/Society of Critical Care Medicine. Chest. 1992 6;101(6):1644–55. 1303622 10.1378/chest.101.6.1644

[24] Bossuyt PM, Reitsma JB, Bruns DE, Gatsonis CA, Glasziou PP, Irwig LM, et al. Towards complete and accurate reporting of studies of diagnostic accuracy: the STARD initiative2003 2003-01-04 08:00:00. 41–4 p. 10.1136/bmj.326.7379.41 PMC1124931 12511463

[25] Mencacci A, Leli C, Cardaccia A, Meucci M, Moretti A, D'Alò F, et al Procalcitonin Predicts Real-Time PCR Results in Blood Samples from Patients with Suspected Sepsis. PloS one. 2012;7(12):e53279 10.1371/journal.pone.0053279 23300907 PMC3531374

[26] Josefson P, Stralin K, Ohlin A, Ennefors T, Dragsten B, Andersson L, et al Evaluation of a commercial multiplex PCR test (SeptiFast) in the etiological diagnosis of community-onset bloodstream infections. European journal of clinical microbiology & infectious diseases: official publication of the European Society of Clinical Microbiology. 2011 9;30(9):1127–34. 10.1007/s10096-011-1201-6 21373774

[27] Yanagihara K, Kitagawa Y, Tomonaga M, Tsukasaki K, Kohno S, Seki M, et al Evaluation of pathogen detection from clinical samples by real-time polymerase chain reaction using a sepsis pathogen DNA detection kit. Critical care (London, England). 2010;14(4):R159. 10.1186/cc9234 PMC2945143 20731880

[28] Schrader C, Schielke A, Ellerbroek L, Johne R. PCR inhibitors—occurrence, properties and removal. Journal of applied microbiology. 2012 11;113(5):1014–26. 10.1111/j.1365-2672.2012.05384.x 22747964

[29] Leli C, Cardaccia A, Ferranti M, Cesarini A, D'Alo F, Ferri C, et al Procalcitonin better than C-reactive protein, erythrocyte sedimentation rate, and white blood cell count in predicting DNAemia in patients with sepsis. Scand J Infect Dis. 2014 11;46(11):745–52. 10.3109/00365548.2014.936493 25195647

[30] Charles PE, Ladoire S, Aho S, Quenot J-P, Doise J-M, Prin S, et al Serum procalcitonin elevation in critically ill patients at the onset of bacteremia caused by either gram negative or gram positive bacteria. BMC Infectious Diseases. 2008;8(1):1–8. 18366777 10.1186/1471-2334-8-38 PMC2289831

[31] Leli C, Ferranti M, Moretti A, Al Dhahab ZS, Cenci E, Mencacci A. Procalcitonin Levels in Gram-Positive, Gram-Negative, and Fungal Bloodstream Infections. Disease Markers. 2015;2015:8. 10.1155/2015/701480 PMC4380090 25852221

[32] Yu CW, Juan LI, Hsu SC, Chen CK, Wu CW, Lee CC, et al Role of procalcitonin in the diagnosis of infective endocarditis: a meta-analysis. American Journal of Emergency Medicine. 2013 6;31(6):935–41. 10.1016/j.ajem.2013.03.008 23601504

[33] Sulyok I, Fleischmann E, Stift A, Roth G, Lebherz-Eichinger D, Kasper D, et al Effect of preoperative fever-range whole-body hyperthermia on immunological markers in patients undergoing colorectal cancer surgery. Br J Anaesth. 2012 11;109(5):754–61. 10.1093/bja/aes248 22855633

[34] Molter GP, Soltesz S, Kottke R, Wilhelm W, Biedler A, Silomon M. Procalcitonin plasma concentrations and systemic inflammatory response following different types of surgery. Anaesthesist. 2003 3;52(3):210–7. 12666002 10.1007/s00101-003-0460-8

[35] Ataoğlu H, Yilmaz F, Uzunhasan I, Çetin F, Temiz L, Döventaş Y, et al Procalcitonin: A Novel Cardiac Marker with Prognostic Value in Acute Coronary Syndrome. Journal of International Medical Research. 2010 2 1, 2010;38(1):52–61. 10.1177/147323001003800106 20233513

[36] Conti G, Amore A, Chiesa M, Mancuso D, Cirina P, Mengozzi G, et al Procalcitonin as a marker of micro-inflammation in hemodialysis. J Nephrol. 2005 May-Jun;18(3):282–8. 16013016

[37] Patout M, Salaun M, Brunel V, Bota S, Cauliez B, Thiberville L. Diagnostic and prognostic value of serum procalcitonin concentrations in primary lung cancers. Clin Biochem. 2014 12;47(18):263–7. 10.1016/j.clinbiochem.2014.09.002 25218831

[38] Machens A, Lorenz K, Dralle H. Utility of serum procalcitonin for screening and risk stratification of medullary thyroid cancer. The Journal of clinical endocrinology and metabolism. 2014 8;99(8):2986–94. 10.1210/jc.2014-1278 24840813

[39] Wacker C, Prkno A, Brunkhorst FM, Schlattmann P. Procalcitonin as a diagnostic marker for sepsis: a systematic review and meta-analysis. The Lancet Infectious diseases. 2013 5;13(5):426–35. 10.1016/S1473-3099(12)70323-7 23375419

[40] Chuang YC, Chang SC, Wang WK. Using the rate of bacterial clearance determined by real-time polymerase chain reaction as a timely surrogate marker to evaluate the appropriateness of antibiotic usage in critical patients with Acinetobacter baumannii bacteremia. Critical care medicine. 2012 8;40(8):2273–80. 10.1097/CCM.0b013e3182515190 22809902

[41] Tschaikowsky K, Hedwig-Geissing M, Schmidt J, Braun GG. Lipopolysaccharide-Binding Protein for Monitoring of Postoperative Sepsis: Complemental to C-Reactive Protein or Redundant? PLoS ONE 6(8): e23615 10.1371/journal.pone.0023615 21901123 PMC3161994

[42] Lichtenstern C, Brenner T, Bardenheuer HJ, Weigand MA. Predictors of survival in sepsis: what is the best inflammatory marker to measure? Current opinion in infectious diseases. 2012 6;25(3):328–36. 10.1097/QCO.0b013e3283522038 22421751

[43] Vincent JL, Brealey D, Libert N, Abidi NE, O'Dwyer M, Zacharowski K, et al Rapid Diagnosis of Infection in the Critically Ill, a Multicenter Study of Molecular Detection in Bloodstream Infections, Pneumonia, and Sterile Site Infections. Critical care medicine. 2015 11;43(11):2283–91. 10.1097/CCM.0000000000001249 26327198 PMC4603364

[44] Bacconi A, Richmond GS, Baroldi MA, Laffler TG, Blyn LB, Carolan HE, et al Improved Sensitivity for Molecular Detection of Bacterial and Candida Infections in Blood. Journal of clinical microbiology. 2014 9 1, 2014;52(9):3164–74. 10.1128/JCM.00801-14 PMC4313132 24951806

[45] Idelevich EA, Silling G, Niederbracht Y, Penner H, Sauerland MC, Tafelski S, et al Impact of multiplex PCR on antimicrobial treatment in febrile neutropenia: a randomized controlled study. Medical microbiology and immunology. 2015 10;204(5):585–92. 10.1007/s00430-014-0385-7 25573349

[46] Tafelski S, Nachtigall I, Adam T, Bereswill S, Faust J, Tamarkin A, et al Randomized controlled clinical trial evaluating multiplex polymerase chain reaction for pathogen identification and therapy adaptation in critical care patients with pulmonary or abdominal sepsis. The Journal of international medical research. 2015 6;43(3):364–77. 10.1177/0300060514561135 25911587

[47] Hranjec T, Rosenberger LH, Swenson B, Metzger R, Flohr TR, Politano AD, et al Aggressive versus conservative initiation of antimicrobial treatment in critically ill surgical patients with suspected intensive-care-unit-acquired infection: a quasi-experimental, before and after observational cohort study. The Lancet Infectious diseases. 2012 10;12(10):774–80. 10.1016/S1473-3099(12)70151-2 22951600 PMC3462590

[48] Dierkes C, Ehrenstein B, Siebig S, Linde HJ, Reischl U, Salzberger B. Clinical impact of a commercially available multiplex PCR system for rapid detection of pathogens in patients with presumed sepsis. BMC infectious diseases. 2009;9:126 10.1186/1471-2334-9-126 19671147 PMC2739209

[49] Avolio M, Diamante P, Modolo ML, De Rosa R, Stano P, Camporese A. Direct molecular detection of pathogens in blood as specific rule-in diagnostic biomarker in patients with presumed sepsis: our experience on a heterogeneous cohort of patients with signs of infective systemic inflammatory response synsdrome Shock (Augusta, Ga). 2014;42(2):86–92. 10.1097/SHK.0000000000000191 24727869

