# Supplementary material for: Evaluation of the Septifast MGrade Test on Standard Care Wards—A Cohort Study
Source: PLoS One. 2016 Mar 17;11(3):e0151108. doi: 10.1371/journal.pone.0151108 (PMC4795709; doi:10.1371/journal.pone.0151108)
Supplement: S1 Table — (DOCX) [file pone.0151108.s001.docx]

**S1 Table: Distribution of infections according to ECDC classification criteria**. Type = type of infection, modified ECDC class according to (19);

| Type | ECDC class | n | % |
| --- | --- | --- | --- |
| Bloodstream infection^1^ | C-CVC^3^ (n=8), S-DIG^4^ (n=12), S-PUL^5^ (n=4), S-SSI^6^ (n=2), S-SST^7^ (n=3) S-UTI^8^ (n=5), S-OTH^9^ (n=12), S-UO^10^ (n=10) | 56 | 37.6% |
| Respiratory tract infection^2^ | PN1^11^ (n=2), PN3^12^ (n=2), PN5^13^ (n=34), LRI-Bron^14^(n=1), LRI-Lung^15^(n=1) | 40 | 26.9% |
| Gastrointestinal system infection^2^ | GI-CDI^16^ (n=1), GI-GE^17^ (n=3), GI-IAB^18^ (n=4), EENT-ORAL^19^ (n=1) | 9 | 6% |
| Urinary tract infection^2^ | UTI-A^20^ (n=10), UTI-B^21^ (n=7) | 17 | 11.4% |
| Others^2^ | SYS-CESP^22^ (n=2), SYS-DI^23^ (n=5), SSI-S^24^ (n=5), SSI-D^25^ (n=2), CVS-Card^26^ (n=1), CVS-Vasc^27^ (n=1), SST-Skin^28^ (n=2), SST-ST^29^ (n=3), REPR-OREP^30^ (n=2), CRI2-CVC^31^(n=1), CNS-IC^32^ (n=1), CNS-SA^33^(n=1), BJ-JNT^34^ (n=1) | 27 | 18.1% |
| Total |  | 149 | 100% |

^1^= blood culture positive ^2^= blood culture negative, ^3^= blood stream infection (BSI), related to central vascular catheter; ^4^= BSI, secondary digestive tract infection; ^5^= BSI, secondary to pulmonary infection; ^6^= BSI, secondary to surgical site infection; ^7^= BSI, secondary to skin and soft tissue infection; ^8^= BSI, secondary to urinary tract infection; ^9^= BSI, secondary to another infection; ^10^= BSI, (confirmed) unknown origin; ^11^= pneumonia, positive quantitative culture from minimally contaminated lower respiratory tract specimen; ^12^= pneumonia, positive sputum culture or non-quantitative culture from lower respiratory tract specimen; ^13^= pneumonia, clinical signs of pneumonia without positive microbiology; bronchitis, tracheobronchitis, bronchiolitis, tracheitis, without evidence of pneumonia; ^14^= LRI, other infections of the lower respiratory tract, bronchitis, tracheobronchitis, bronchiolitis, tracheitis; ^15^= lower respiratory tract infection, other than pneumonia;^16^= gastrointestinal system infections (GI) *clostridium difficile* infection; ^17^= GI, gastroenteritis (excluding CDI); ^18^= GI, intra-abdominal, not specified elsewhere; ^19^= eye, ear, nose or mouth infection (EENT), oral cavity (mouth, tongue, or gums); ^20^= urinary tract infection (UTI), microbiologically confirmed symptomatic UTI; ^21^= UTI, not microbiologically confirmed symptomatic UTI; ^22^= systemic infections (SYS), clinical sepsis in adults and children; ^23^= SYS, disseminated infection; ^24^= surgical site infection (SSI), superficial incisional; ^25^= surgical site infection (SSI), deep incisional;^26^= cardiovascular system infection (CVS), myocarditis or pericarditis; ^27^ = CVS, arterial or venous infection; ^28^= skin and soft tissue infections (SST), skin; ^29^= SST, soft tissue (necrotizing fasciitis, infectious gangrene, necrotizing cellulitis, infectious myositis, lymphadenitis, or lymphangitis); ^30^= reproductive tract infections (REPR) – other infections of the male or female reproductive tract (OREP), ^31^= central vascular catheter-related infection (CRI), general CVC-related infection (no positive blood culture); ^32^= central nervous system infection (CNS), intracranial infection; ^33^= CNS-SA: spinal abscess without meningitis; ^34^BJ-JNT= joint or bursa infections
