# Supplementary material for: Evaluation of the Septifast MGrade Test on Standard Care Wards—A Cohort Study
Source: PLoS One. 2016 Mar 17;11(3):e0151108. doi: 10.1371/journal.pone.0151108 (PMC4795709; doi:10.1371/journal.pone.0151108)
Supplement: S2 Table — (DOCX) [file pone.0151108.s002.docx]

**S2 Table: Comparison between BC and SF analysis**

| Pathogen | | SF | BC |
| --- | --- | --- | --- |
| Gram-positive bacteria | |  |  |
|  | *Staphylococcus aureus* | 9 | 9 |
|  | Coagulases-negative *Staphylococci*^1^ | 9 | 11 |
|  | *Streptococcus species^2^* | 7 | 4 |
|  | *Enterococcus faecium* | 3 | 1 |
|  | *Enterococcus faecalis* | 3 | 2 |
|  | *Bacillus species* | 0 | 1 |
|  | *Corynebacterium species* | 0 | 1 |
| Gram-negative bacteria | |  |  |
|  | *Escherichia coli* | 6 | 11 |
|  | *Klebsiella pneumoniae/oxytoca* | 8 | 3 |
|  | *Raoultella ornithinolytica* | 0 | 1 |
|  | *Pseudomonas aeruginosa* | 5 | 5 |
|  | *Enterobacter cloacae/aerogenes* | 3 | 1 |
|  | *Citrobacter freundii* | 0 | 1 |
|  | *Citrobacter koseri* | 0 | 1 |
|  | *Bacteroides thetaitaomicron* | 0 | 1 |
| Fungal | |  |  |
|  | Candida albicans | 1 | 1 |

^1^*Staphylococcus epidermidis* (BC: n=10), *Staphylococcus haemolyticus* (BC: n=1), ^2^*Streptococcus pneumoniae* (SF: n=3), *Streptococcus pyogenes* (SF: n=1), *Streptococcus species* (SF n=3), *Streptococcus constellatus (BC: n=1), Streptococcus mitis/oralis* (BC: n=1), *Streptococcus mutans* (BC: n=1*),* group A β-hemolytic streptococcus (BC: n=1)
