# Supplementary material for: Evaluation of the Septifast MGrade Test on Standard Care Wards—A Cohort Study
Source: PLoS One. 2016 Mar 17;11(3):e0151108. doi: 10.1371/journal.pone.0151108 (PMC4795709; doi:10.1371/journal.pone.0151108)
Supplement: S3 Table — (DOCX) [file pone.0151108.s003.docx]

**S3 Table: BC negative patients with detection of pathogens in the SF test**

| # | ECDC | SF result | Comment |
| --- | --- | --- | --- |
| 1 | S-DIG | *K. pneumoniae/ oxytoca* | Cholecystitis, two pairs of BCs and urine culture remained negative |
| 2 | CNS-MEN | *S. pneumoniae* | Meningitis, CFS fluid culture: *S. pneumoniae* |
| 3 | S-DIG* | *E. faecium* | Pancreatitis, BC and urine culture negative, stool culture without pathological pathogens |
| 4 | S-DIG | *K. pneumoniae/ oxytoca* | Pancreatitis, secondary sclerosing cholangitis, two sets of BCs remaind negative |
| 5 | S-DIG | *E. faecium* | Colon perforation, one set of BCs remained negative |
| 6 | S-PUL | *P. aeruginosa* | Pneumonia, two sets of BCs remained negative |
| 7 | S-DIG | *K. pneumoniae/ oxytoca* | Cholecystitis, liver abscess, abscess culture: K. *pneumoniae* and *E. faecium* |
| 8 | S-PUL | *S. pneumoniae* | Pneumonia, two sets of BC remained negative |
| 9 | S-SST | *K. pneumoniae/ oxytoca* | Perforation and abscess in the sigmoid colon, abscess culture: *K. pneumonia* |
| 10 | S-OTH* | *Streptococcus species* | Endocarditis, negative BC analyses, response to therapy with penicillin G |
| 11 | S-PUL | *K. pneumoniae/ oxytoca* | Pneumonia, radiological confirmation, seven BCs sets remained negative |
| 12 | S-PUL | *E. cloace/aerogenes* | ALL/NHL, before: pneumonia/ARDS/ECCMO, two BC sets remained negative |
| 13 | S-OTH* | *S. aureus*, CoNS | Endocarditis, six BC sets remained negative |
| 14 | S-UO | *S. aureus* | NSCLC, four BC sets remained negative, response to therapy: ampicillin and enzyme inhibitor |

*antibiotic therapy started before BC sampling, ECDC= modified ECDC class according to (19)
