# Supplementary material for: Evaluation of the Septifast MGrade Test on Standard Care Wards—A Cohort Study
Source: PLoS One. 2016 Mar 17;11(3):e0151108. doi: 10.1371/journal.pone.0151108 (PMC4795709; doi:10.1371/journal.pone.0151108)
Supplement: S4 Table — (DOCX) [file pone.0151108.s004.docx]

**S4 Table: Patients with detected pathogens and <0.37ng/ml PCT levels;** ECDC= modified ECDC class according to (19).

| # | ECDC | SF result | BC result | PCT | CRP | Comment |
| --- | --- | --- | --- | --- | --- | --- |
| 1 | BSI-C-CVC | negative | *S. epidermidis*  (14hrs*) | 0.11 | 9.29 | central line infection, rectal cancer, Hepatitis C infection, two positive BC sets |
| 2 | BSI-UO | *S. aureus* | *S. aureus*  (31.5hrs*^+^) | 0.36 | 10.25 | aortic aneurysm with a pseudoaneurysm (suspected infection focus), six BC sets positive |
| 3 | C-CVC | CoNS | *S. epidermidis*  (19hrs*) | 0.1 | 7.36 | central line infection, B-cell lymphoma, severe neutropenia (chemotherapy related), six positive BC sets |
| 4 | CRI3-CVC | CoNS | *S. epidermidis*  *(20hrs*)* | 0.16 | 4.26 | central line infection, ten BC sets positive, after major poly-trauma with a spleen and liver rupture, multiple bone fracture, multiple surgical interventions |
| 5 | S-OTH | *Streptococcus*  *species* | *S. mitis/oralis*  (17hrs*) | 0.28 | 3.64 | endocarditis (histological confirmation) seven BC sets positive |
| 6 | S-OTH | *Streptococcus*  *species* | negative^+^ | 0.16 | 6.04 | endocarditis (histological confirmation), six sets of BC negative, response to therapy with penicillin G |
| 7 | S-OTH | negative | *P. aeruginosa*  (17hrs*) | 0.35 | 24.27 | acute renal failure, cardiomyopathy, Left Ventricular Assist Device (LVAD) implementation, two positive BC sets. |
| 8 | S-OTH | negative | *S. mutans*  (41hrs*) | 0.06 | 7.17 | endocarditis, sixteen positive BC sets; very bad dental chart, |
| 9 | S-PUL | *S. pneumonie* | negative | 0.16 | 14.68 | pneumonia, radiological confirmation, two sets of BC remained negative |
| 10 | S-PUL | *K. pneumoniae/ oxytoca* | negative | 0.17 | 31.92 | pneumonia, radiological confirmation, six sets of BC analyses remained negative, Morbus Hodgkin |
| 11 | SSI-S | *S. aureus* | *S. aureus*  (8.5 hrs*) | 0.35 | 15.4 | colon cancer, surgical wound infections, surgical revision, one BC analysis conducted, positive result |
| 12 | S-SST | *E. cloace/ aerogenes* | negative | 0.15 | 8.72 | T-cell lymphoma (ALCL), ARDS, wound swabs with *Pseudomonas aeruginosa*, *Enterococcus species* |
| 13 | S-UO | *S. aureus* | negative | 0.05 | 10.49 | non-small cell lung cancer, unclear infection focus, response to therapy: ampicillin and enzyme inhibitor |

*time to positivity in hrs, ^+^antimicrobial therapy was started before BC sampling, PCT is given in ng/ml, CRP is given in mg/dl
